# Supplementary material for: Secular Trend of Self-Concept in the Context of Childhood Obesity—Data from the CHILT III Programme, Cologne
Source: Children (Basel). 2023 Jan 7;10(1):127. doi: 10.3390/children10010127 (PMC9856785; doi:10.3390/children10010127)
Supplement: Supplementary file 1 [file children-10-00127-s001.zip › children-2135346-supplementary.pdf]

Supplemental materials

Titel: Secular trend of psychological distress in the context of childhood obesity - data from the CHILT III programme, Cologne

Authors: Lisa Grünberg[1], Nina Eisenburger[1], Nina Ferrari[1,2], David Friesen[1], Fabiola Haas[1], Marlen Klaudius[1], Lisa Schmidt[1], Christine Joisten[1,2]

[1] Department for Physical Activity in Public Health, Institute of Movement and Neurosciences, German Sport University Cologne, Am Sportpark Müngersdorf 6, 50933 Cologne, Germany
[2] Cologne Center for Prevention in Childhood and Youth/Heart Center Cologne, University Hospital of Cologne, Kerpener Str. 62, 50937 Cologne, Germany

Included supplemental materials:

Table S1-S3

Table S1: Descriptive statistics of all time clusters separated by sex

**Overall** (boys *n* = 133) (girls *n* = 129)	**2005-2006** (boys *n* = 15) (girls *n* = 25)	**2007-2008** (boys *n* = 19) (girls *n* = 19)	**2009-2011** (boys *n* = 14) (girls *n* = 20)	**2012-2014** (boys *n* = 22) (girls *n* = 20)	**2015-2017** (boys *n* = 20) (girls *n* = 25)	**2018-2020** (boys *n* = 23) (girls *n* = 20)	***p-value***
**(1) sex n (%)**							
**male**	15 (37.5%)	19 (50.0%)	14 (41.2%)	22 (52.4%)	20 (44.4%)	23 (53.5%)	
**female**	25 (62.5%)	19 (50.0%)	20 (58.8%)	20 (47.6%)	25 (55.6%)	20 (46.5%)	
**(2) age (in years)**							
**boys**	12.2 ± 2.5 (15)	13.3 ± 1.8 (19)	12.3 ± 1.9 (14)	13.0 ± 2.0 (22)	12.6 ± 2.5 (20)	12.8 ±1.7 (23)	.632†
**girls**	12.3 ± 1.9 (25)	12.6 ± 2.2 (19)	12.9 ± 1.5 (20)	11.8 ± 3.0 (20)	12.3 ± 2.1 (25)	12.0 ± 2.2 (20)	.626†
**(3) height (in m)**							
**boys**	1.56 ± 0.13 (15)	1.66 ± 0.12 (19) ‡	1.58 ± 0.11 (14)	1.65 ± 0.11 (22) ‡	1.57 ± 0.14 (20)	1.62 ± 0.12 (23)	.144†
**girls**	1.57 ± 0.09 (25)	1.57 ± 0.12 (19)	1.56 ± 0.07 (20)	1.52 ± 0.09 (20)	1.55 ± 0.10 (25)	1.58 ± 0.09 (20)	.506†
**(4) weight (in kg)**							
**boys**	79.9 ± 24.7 (15)	83.5 ± 22.0 (19)	72.5 ± 18.0 (14)	87.5 ± 16.5 (22) ‡	78.3 ± 27.2 (20)	82.6 ± 23.7 (23)	.483†
**girls**	71.2 ± 14.8 (25)	72.9 ± 17.9 (19)	74.6 ± 12.9 (20)	67.1 ± 14.7 (20)	71.3 ± 16.7 (25)	76.3 ± 17.8 (20)	.547†
**(5) BMI (in kg/m²)**							
**boys**	30.6 ± 5.6 (15)	29.8 ± 4.7 (19)	28.6 ± 4.2 (14)	32.1 ± 4.6 (22) ‡	31.1 ± 7.5 (20)	30.8 ± 5.0 (23)	.525†
**girls**	28.8 ± 4.3 (25)	29.0 ± 4.1 (19)	30.5 ± 3.9 (20)	28.6 ± 3.7 (20)	29.2 ± 4.6 (25)	30.1 ± 4.4 (20)	.621†
**(6) BMI-SDS**							
**boys**	2.53 ± 0.45 (15)	2.32 ± 0.41 (19)	2.28 ± 0.51 (14)	2.57 ± 0.51 (22)	2.49 ± 0.48 (20)	2.44 ± 0.47 (23)	.397†
**girls**	2.34 ± 0.53 (25)	2.41 ± 0.39 (19)	2.51 ± 0.41 (20)	2.46 ± 0.46 (20)	2.43 ± 0.44 (25)	2.58 ± 0.41 (20)	.559†

overall (boys n = 97) (girls n = 108)	2005-2006 (boys n = 13) (girls n = 18)	2007-2008 (boys n = 17) (girls n = 19)	2009-2011 (boys n = 14) (girls n = 15)	2012-2014 (boys n = 15) (girls n = 15)	2015-2017 (boys n = 16) (girls n = 23)	2018-2020 (boys n = 22) (girls n = 18)	
**(7) migration background (yes)**							
**boys**	1 (7.7%)	1 (5.9%)	1 (7.1%)	3 (20.0%)	1 (6.3%)	3 (13.6%)	.749#
**girls**	1 (5.6%)	2 (10.5%)	0 (0.0%)	3 (20.0%)	2 (8.7%)	5 (27.8%)	.148#
**(8) media consumption (in h/day)**							
**boys**	1.63 ± 0.75 (13)	1.74 ± 0.96 (17)	0.89 ± 0.55 (14) ‡	1.39 ± 0.88 (15)	6.52 ± 4.69 (16)	6.65 ± 4.42 (22)	<.001†
**girls**	2.10 ± 1.30 (18)	1.60 ± 0.98 (19)	1.77 ± 1.05 (15)	1.49 ± 0.92 (15)	4.37 ± 2.47 (23)	6.05 ± 3.81 (18)	<.001†

The data are presented as the mean ± SD; BMI = body mass index; BMI SDS = Body mass index standard deviation score; p-values calculated with the † analysis of variance (ANOVA); ‡ unpaired t-test, # Chi square test; $p < 0.05$;

Table S2: Results analysing differences in the domains of self-concept over time separated by sex

	periods of time							
	**Overall** (boys n = 133) (girls n = 129)	**2005-2006** (boys n = 15) (girls n = 25)	**2007-2008** (boys n = 19) (girls n = 19)	**2009-2011** (boys n = 14) (girls n = 20)	**2012-2014** (boys n = 22) (girls n = 20)	**2015-2017** (boys n = 20) (girls n = 25)	**2018-2020** (boys n = 23) (girls n = 20)	*p-value*†
**(1) physical appearance**								
**boys**	55.8 ± 15.4	50.0 ± 14.7	52.7 ± 19.3	57.7 ± 11.4	54.2 ± 14.3	59.8 ± 15.7	59.2 ± 15.0	.269
**girls**	53.5 ± 15.9	49.4 ± 14.6	50.2 ± 11.9	57.4 ± 13.2	57.1 ± 17.4	54.0 ± 17.9	53.5 ± 18.8	.220
**(2) social competence**								
**boys**	75.4 ± 18.8	75.6 ± 19.2	70.6 ± 23.1	80.4 ± 21.5	72.8 ± 16.9	73.5 ± 19.7	80.5 ± 12.9	.505
**girls**	76.5 ± 18.4	78.4 ± 19.5	80.9 ± 19.4	78.0 ± 16.6	79.0 ± 13.1	71.3 ± 19.1	72.8 ± 21.0	.519
**(3) scholastic competence**								
**boys**	75.6 ± 16.7	78.9 ± 18.4	76.6 ± 14.2	82.2 ± 16.9	72.0 ± 17.0	71.9 ± 14.3	75.2 ± 19.1	.414
**girls**	74.9 ± 16.5	72.3 ± 20.1	78.7 ± 15.6	77.1 ± 12.7	74.8 ± 14.8	72.4 ± 17.3	75.4 ± 17.5	.757
**(4) behavioral conduct**								
**boys**	73.5 ± 16.7	75.8 ± 18.0	70.9 ± 18.5	83.3 ± 16.3	73.1 ± 16.8	67.5 ± 13.6	73.5 ± 15.8	.219
**girls**	74.5 ± 15.9	74.3 ± 16.9	78.1 ± 14.6	77.6 ± 14.9	70.6 ± 18.8	74.4 ± 13.4	72.4 ± 17.1	.579
**(5) global self worth**								
**boys**	71.9 ± 15.9	76.3 ± 13.0	69.9 ± 16.6	80.7 ± 13.6	67.4 ± 14.9	68.2 ± 18.1	72.9 ± 15.9	.219
**girls**	73.4 ± 16.1	72.6 ± 18.8	69.8 ± 17.6	73.5 ± 11.7	72.5 ± 12.7	75.2 ± 17.9	76.3 ± 16.8	.819

The data are presented as the mean ± SD; p-values are calculated with an † analysis of covariance (ANCOVA) adjusted for age, adolescent status and BMI-SDS

Table S3: Results of the ANCOVA analysing the domains of self-concept adjusted for sex, age, BMI SDS, adolescent status, migration background, media consumption and physical fitness

		periods of time						
	Overall (n = 205)	2005-2006 (n = 31)	2007-2008 (n = 36)	2009-2011 (n = 29)	2012-2014 (n = 30)	2015-2017 (n = 39)	2018-2020 (n = 40)	***p-value***†
(1) physical appearance	54.8 ± 16.0	51.0 ± 15.1	51.4 ± 15.9	59.2 ± 12.3	56.2 ± 17.2	54.7 ± 16.8	56.5 ± 17.0	.466
(2) social competence	76.5 ± 18.4	77.2 ± 17.8	76.4 ± 21.2	81.4 ± 17.8	74.9 ± 15.4	72.6 ± 20.2	77.6 ± 16.7	.476
(3) scholastic competence	76.3 ± 16.2	78.1 ± 17.4	77.7 ± 15.0	80.9 ± 14.5	72.9 ± 16.2	74.0 ± 15.2	74.9 ± 18.4	.534
(4) behavioral conduct	74.7 ± 16.3	77.0 ± 15.9	74.4 ± 17.2	83.1 ± 14.2	70.8 ± 17.4	71.8 ± 13.9	72.8 ± 16.6	.101
(5) global self worth	73.1 ± 16.6	76.0 ± 16.6	69.7 ± 17.2	77.9 ± 13.1	69.0 ± 15.6	72.2 ± 19.0	74.2 ± 16.3	.237

The data are presented as the mean ± SD; p-values are calculated with an † analysis of covariance (ANCOVA) adjusted by sex, age, BMI SDS, adolescent status, migration background, media consumption and physical fitness
